# A proactive multidisciplinary approach to *Naja atra* envenomation reduces surgical burden and length of hospital stay

**DOI:** 10.1590/1678-9199-JVATITD-2026-0029

**Published:** 2026-04-03

**Authors:** Hsien-Po Huang, Ting-Kuang Yeh, Po-Hsiu Huang, Kuo-Lung Lai, Chih-Sheng Lai, Po-Yu Liu, Yan‑Chiao Mao

**Affiliations:** 1 Division of Infectious Diseases, Department of Internal Medicine, Taichung Veterans General Hospital, Taichung, Taiwan.; 2 Division of Allergy, Immunology and Rheumatology, Department of Internal Medicine, Taichung Veterans General Hospital, Taichung, Taiwan.; 3 Division of Plastic and Reconstructive Surgery, Department of Surgery, Taichung Veterans General Hospital, Taichung, Taiwan.; 4 School of Medicine, College of Medicine, National Yang Ming Chiao Tung University, Taipei, Taiwan.; 5 Department of Post-Baccalaureate Medicine, College of Medicine, National Chung Hsing University, Taichung, Taiwan.; 6 Doctoral Program in Translational Medicine, Rong Hsing Translational Medicine Research Center, National Chung Hsing University, Taichung, Taiwan.; 7 PhD Program in Medical Biotechnology, College of Medical Science and Technology, Taipei Medical University, Taipei, Taiwan.; 8 Department of Medical Toxicology, Taichung Veterans General Hospital, Taichung, Taiwan.; 9 College of Medicine, National Defense Medical University, Taipei, Taiwan.

**Keywords:** Cobra envenomation, Multidisciplinary team, Length of stay, Surgical intervention

## Abstract

We report that the implementation of a multidisciplinary team (MDT) protocol - integrating early ultrasound surveillance, prompt surgical intervention, targeted antimicrobial therapy, and optimized antivenom administration - was associated with a marked reduction the length of hospital stay and surgical burden among patients with *Naja atra* envenomation at a tertiary medical center in Taiwan. This approach shifts management from a reactive to a proactive strategy by enabling the early detection of tissue involvement and timely intervention.

## Graphical abstract

**Figure:**
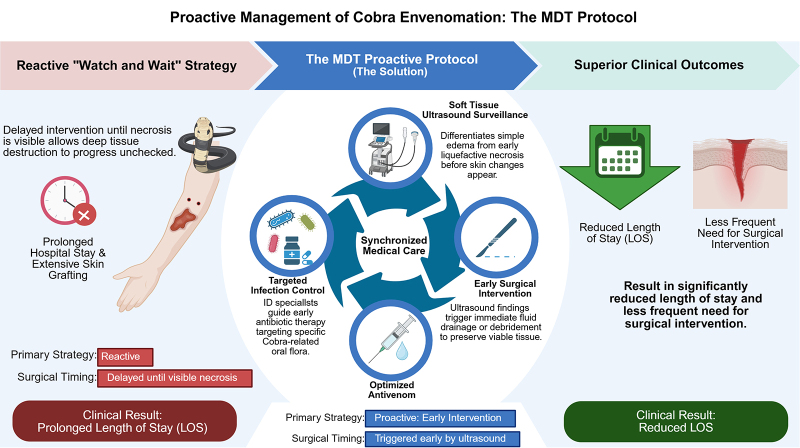

Dear Editor,

Cobra envenomation caused by *Naja atra* in Taiwan is characterized by severe local tissue destruction, frequent wound infection, and a high demand for surgical intervention, despite timely administration of antivenom [1]. Current management strategies remain largely reactive, with surgical intervention often delayed until overt necrosis becomes clinically apparent, thereby allowing progressive tissue damage to evolve.

To address this limitation, we implemented a multidisciplinary team (MDT) protocol integrating early ultrasound surveillance, prompt surgical intervention, targeted antimicrobial therapy, and optimized antivenom administration. This strategy is supported by emerging evidence suggesting that local tissue necrosis in *Naja atra* envenomation is driven by cytotoxins with high tissue affinity, and that necrotic tissue may act as a reservoir of venom that is poorly accessible to circulating antivenom [1]. Early intervention may, therefore, limit ongoing venom-mediated tissue damage and reduce the subsequent surgical burden. The four pillars of the protocol consist of:

1. Soft-tissue *ultrasound monitoring* for the early detection of deep tissue involvement before the onset of overt skin changes. This technique, performed by rheumatologists, enables differentiation between subcutaneous edema (“cobblestone appearance”) and early liquefactive necrosis or abscess formation (discrete fluid collections), allowing for the identification of clinically occult tissue damage.
2. *Early surgical intervention* to eliminate venom-containing necrotic tissue and prevent progression. Ultrasound findings prompt early consultation with plastic surgeons. Identified fluid collections can be promptly drained or debrided, rather than delaying intervention until overt necrosis or gangrene develops.
3. *Targeted infection control* addressing the polymicrobial flora associated with cobra bite wounds. Early involvement of infectious disease specialists allows for the timely initiation of antimicrobial therapy targeting common pathogens (e.g., *Morganella morganii*, *Enterococcus* spp.) [2], thereby reducing the risk of secondary infection and impaired wound healing.
4. *Optimized antivenom administration* to neutralize toxins. Standardized antivenom use, guided by local protocols and supervised by toxicology specialists, facilitates early neutralization of venom and supports overall clinical management.

Between 2013 and 2025, Taichung Veterans General Hospital in Taiwan recorded 184 emergency department visits related to snakebites, of which 79 (43%) were attributed to *Naja atra* envenomation and 53 required hospitalization. Following the implementation of the multidisciplinary protocol, clinical outcomes significantly improved. Among hospitalized patients, a comparison between the initial period (2013-2017) and the integrated care period (2018-2025) showed that the mean length of hospital stay decreased from 27.42 ± 12.39 days to 13.37 ± 7.47 days. The average number of surgical interventions per patient also declined from 4.20 ± 2.35 to 2.00 ± 2.12. Mortality remained at 0%, while limb salvage rates remained high (90.7% vs. 94.4%).

In conclusion, a multidisciplinary and proactive management approach represents a paradigm shift in the management of cytotoxic snakebites, particularly *Naja atra* envenomation, by facilitating earlier intervention, reducing the surgical burden, and improving clinical outcomes.

## Data Availability

Not applicable.
